# Evaluation of bone formation within β-tricalcium phosphate scaffolds in a sheep scapular bioreactor model using micro-computed tomography analysis

**DOI:** 10.1093/rb/rbag097

**Published:** 2026-05-21

**Authors:** D S Abdullah Al Maruf, Matt Darnell, Jiongyu Ren, Eva Tomaskovic-Crook, Kai Cheng, Will Lewin, Hedi V Kruse, Daniel Lawrence, Peilin Luo, Innes K Wise, Aditi Gupta, David Leinkram, Timothy Manzie, Krishnan Parthasarathi, James Wykes, Hubert Low, Cate Froggatt, David R McKenzie, Gordon Wallace, Jeremy M Crook, Jonathan R Clark

**Affiliations:** Central Clinical School, Faculty of Medicine and Health, The University of Sydney, Camperdown, NSW 2006, Australia; Integrated Prosthetics and Reconstruction, Chris O’Brien Lifehouse, Camperdown, NSW 2050, Australia; Integrated Prosthetics and Reconstruction, Chris O’Brien Lifehouse, Camperdown, NSW 2050, Australia; Central Analytical Research Facility, Research Infrastructure, Queensland University of Technology, Brisbane, 4059, Australia; NHMRC Centre of Research Excellence for Applied Innovations in Oral Cancer, Camperdown, NSW 2006, Australia; ARC Training Centre for Cells and Tissue Engineering Technologies, Queensland University of Technology, Brisbane, QLD 4000, Australia; Arto Hardy Family Biomedical Innovation Hub, Chris O’Brien Lifehouse, Camperdown, NSW 2050, Australia; Sarcoma and Surgical Research Centre, Chris O’Brien Lifehouse, Camperdown, NSW 2050, Australia; School of Medical Sciences, Faculty of Medicine and Health, The University of Sydney, Camperdown, NSW 2006, Australia; Faculty of Engineering and Information Sciences, Intelligent Polymer Research Institute, AIIM Facility, University of Wollongong, Fairy Meadow, NSW 2519, Australia; Royal Prince Alfred Institute of Academic Surgery, Sydney Local Health District, Camperdown, NSW 2050, Australia; Arto Hardy Family Biomedical Innovation Hub, Chris O’Brien Lifehouse, Camperdown, NSW 2050, Australia; Sarcoma and Surgical Research Centre, Chris O’Brien Lifehouse, Camperdown, NSW 2050, Australia; School of Medical Sciences, Faculty of Medicine and Health, The University of Sydney, Camperdown, NSW 2006, Australia; Arto Hardy Family Biomedical Innovation Hub, Chris O’Brien Lifehouse, Camperdown, NSW 2050, Australia; Sarcoma and Surgical Research Centre, Chris O’Brien Lifehouse, Camperdown, NSW 2050, Australia; School of Physics, The University of Sydney, Camperdown, NSW 2006, Australia; Australian National Fabrication Facility-Materials Node, AIIM Facility, University of Wollongong, NSW 2519, Wollongong, Australia; Integrated Prosthetics and Reconstruction, Chris O’Brien Lifehouse, Camperdown, NSW 2050, Australia; Laboratory Animal Services, The University of Sydney, Camperdown, NSW 2050, Australia; Central Clinical School, Faculty of Medicine and Health, The University of Sydney, Camperdown, NSW 2006, Australia; Integrated Prosthetics and Reconstruction, Chris O’Brien Lifehouse, Camperdown, NSW 2050, Australia; Central Clinical School, Faculty of Medicine and Health, The University of Sydney, Camperdown, NSW 2006, Australia; Integrated Prosthetics and Reconstruction, Chris O’Brien Lifehouse, Camperdown, NSW 2050, Australia; Department of Head and Neck Surgery, Sydney Head and Neck Cancer Institute, Chris O’Brien Lifehouse, Camperdown, NSW 2050, Australia; Central Clinical School, Faculty of Medicine and Health, The University of Sydney, Camperdown, NSW 2006, Australia; Integrated Prosthetics and Reconstruction, Chris O’Brien Lifehouse, Camperdown, NSW 2050, Australia; NHMRC Centre of Research Excellence for Applied Innovations in Oral Cancer, Camperdown, NSW 2006, Australia; Department of Head and Neck Surgery, Sydney Head and Neck Cancer Institute, Chris O’Brien Lifehouse, Camperdown, NSW 2050, Australia; Central Clinical School, Faculty of Medicine and Health, The University of Sydney, Camperdown, NSW 2006, Australia; Integrated Prosthetics and Reconstruction, Chris O’Brien Lifehouse, Camperdown, NSW 2050, Australia; Department of Head and Neck Surgery, Sydney Head and Neck Cancer Institute, Chris O’Brien Lifehouse, Camperdown, NSW 2050, Australia; Central Clinical School, Faculty of Medicine and Health, The University of Sydney, Camperdown, NSW 2006, Australia; Integrated Prosthetics and Reconstruction, Chris O’Brien Lifehouse, Camperdown, NSW 2050, Australia; Department of Head and Neck Surgery, Sydney Head and Neck Cancer Institute, Chris O’Brien Lifehouse, Camperdown, NSW 2050, Australia; Central Clinical School, Faculty of Medicine and Health, The University of Sydney, Camperdown, NSW 2006, Australia; Integrated Prosthetics and Reconstruction, Chris O’Brien Lifehouse, Camperdown, NSW 2050, Australia; Department of Head and Neck Surgery, Sydney Head and Neck Cancer Institute, Chris O’Brien Lifehouse, Camperdown, NSW 2050, Australia; Integrated Prosthetics and Reconstruction, Chris O’Brien Lifehouse, Camperdown, NSW 2050, Australia; Department of Head and Neck Surgery, Sydney Head and Neck Cancer Institute, Chris O’Brien Lifehouse, Camperdown, NSW 2050, Australia; Arto Hardy Family Biomedical Innovation Hub, Chris O’Brien Lifehouse, Camperdown, NSW 2050, Australia; Sarcoma and Surgical Research Centre, Chris O’Brien Lifehouse, Camperdown, NSW 2050, Australia; School of Physics, The University of Sydney, Camperdown, NSW 2006, Australia; NHMRC Centre of Research Excellence for Applied Innovations in Oral Cancer, Camperdown, NSW 2006, Australia; Faculty of Engineering and Information Sciences, Intelligent Polymer Research Institute, AIIM Facility, University of Wollongong, Fairy Meadow, NSW 2519, Australia; NHMRC Centre of Research Excellence for Applied Innovations in Oral Cancer, Camperdown, NSW 2006, Australia; Arto Hardy Family Biomedical Innovation Hub, Chris O’Brien Lifehouse, Camperdown, NSW 2050, Australia; Sarcoma and Surgical Research Centre, Chris O’Brien Lifehouse, Camperdown, NSW 2050, Australia; School of Medical Sciences, Faculty of Medicine and Health, The University of Sydney, Camperdown, NSW 2006, Australia; Faculty of Engineering and Information Sciences, Intelligent Polymer Research Institute, AIIM Facility, University of Wollongong, Fairy Meadow, NSW 2519, Australia; Central Clinical School, Faculty of Medicine and Health, The University of Sydney, Camperdown, NSW 2006, Australia; Integrated Prosthetics and Reconstruction, Chris O’Brien Lifehouse, Camperdown, NSW 2050, Australia; NHMRC Centre of Research Excellence for Applied Innovations in Oral Cancer, Camperdown, NSW 2006, Australia; Royal Prince Alfred Institute of Academic Surgery, Sydney Local Health District, Camperdown, NSW 2050, Australia; Department of Head and Neck Surgery, Sydney Head and Neck Cancer Institute, Chris O’Brien Lifehouse, Camperdown, NSW 2050, Australia

**Keywords:** SP-βTCP, bioreactor chambers, bone regeneration, ADSCs, ovine model

## Abstract

Critical-sized bone defects in load-bearing regions remain a major clinical challenge. This study investigated how β-tricalcium phosphate (βTCP) scaffold pore size and stem cell source influence bone regeneration using a laser-sintered, plasma-treated polyetherketone (P-PEK) dual-chamber scapular bioreactor model in sheep. On the left scapula, selectively polymerized βTCP (SP-βTCP) scaffolds with two pore sizes were implanted for 16 weeks: a Large Pore design (1.875-mm unit cell; 0.93-mm pore) and a Small Pore design (1.5-mm unit cell; 0.66-mm pore). Adjacent Large Pore scaffolds were loaded with either gelatine methacryloyl (GelMA) alone or GelMA encapsulating autologous adipose-derived stem cells (ADSCs). On the right scapula, Large Pore scaffolds containing GelMA-encapsulated autologous or allogeneic ADSCs were implanted for 12 weeks. Bone formation was quantified by micro-computed tomography (µCT) and validated by resin-embedded histology. Small Pore scaffolds generated greater bone volume than Large Pore scaffolds. Autologous ADSC-laden scaffolds outperformed GelMA-only controls, particularly in lower bioreactors interfacing with native bone. No significant differences were detected between autologous and allogeneic ADSCs. Histology confirmed bioreactor-dependent variation, with lower bone-contacting chambers consistently producing more mineralized tissue. These findings highlight the interplay among scaffold architecture, cell source and anatomical niche in optimizing translational bone regeneration.

## Graphical abstract

**Graphical Abstract:**
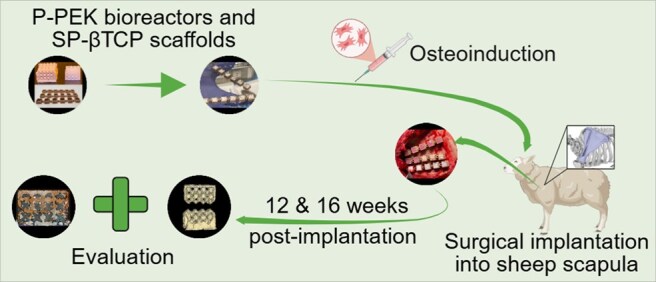

## Introduction

Bone tissue engineering (BTE) holds immense promise for addressing critical-sized bone defects, particularly where both structural integrity and functional restoration are vital for improving patients’ quality of life. Segmental bone defects, where there is discontinuity of the affected bone, caused by trauma, tumour excision or congenital abnormalities, present unique challenges due to the mechanical requirements for load bearing. Autologous bone grafts augmented by titanium plates remain the gold standard reconstructive option for segmental bone defects, but they are constrained by limited donor site availability, associated morbidity and potential complications at the harvest site and plate loosening or stress shielding, which can hinder bone healing [1–3]. As a result, BTE strategies are being explored as clinically viable solutions for repairing critical-sized bone defects by combining osteoinductive and osteoconductive mechanobiological properties with biocompatible materials [4].

In BTE, scaffolds play a pivotal role, ideally providing structural support and a conducive environment for cell attachment, proliferation and differentiation [5, 6]. An essential factor in scaffold design is pore size, as it influences cell infiltration, vascularization and tissue integration. Larger pores (200–500 μm) promote osteoblast and blood vessel infiltration, enhancing vascularization, while smaller pores (50–200 μm) foster cell attachment and osteogenic differentiation, aiding mineralized bone tissue formation [5, 7]. As described in these studies, the underlying mechanisms are attributed to differences in surface area and nutrient diffusion gradients, where smaller pores enhance cell-matrix interactions, while larger pores facilitate angiogenic ingrowth and nutrient transport, together dictating the balance between osteoconduction and vascularization within the scaffold. However, achieving an optimal balance in pore size distribution remains challenging. Among scaffold materials, β-tricalcium phosphate (βTCP) stands out for its osteoconductive properties, supporting bone cell infiltration and growth while resorbing gradually to enable new bone integration. Studies have demonstrated βTCP’s compatibility with both ectopic and orthotopic bone growth, making it a popular choice [6]. Compared to hydroxyapatite (HA), which exhibits higher crystallinity and slower degradation, βTCP resorbs more rapidly, supporting ongoing bone remodelling and the release of calcium and phosphate ions that contribute to mineralized tissue formation [8]. In contrast, bioactive glasses can enhance cellular responses through ionic dissolution products but are often limited by brittle mechanical behaviour and handling challenges [9]. Polymeric scaffolds such as poly(lactic-co-glycolic acid) (PLGA) offer tuneable degradation rates and mechanical flexibility; however, they generally lack intrinsic osteoconductivity unless combined with bioactive ceramic phases [10, 11]. Composite scaffolds integrating polymers with bioactive ceramics have therefore been developed to address these limitations, yet βTCP remains a clinically established material due to its predictable degradation profile and its capacity to support vascularized bone ingrowth in both load-sharing and non-load-bearing applications [10].

βTCP is not freely soluble under physiological conditions and necessitates active resorption by osteoclastic acidification to render its calcium bioavailable for bone regeneration. This occurs when osteoclasts form a sealed zone and pump acid into it via vacuolar-type H^+^-ATPases, dissolving the βTCP crystals [12, 13]. They also secrete enzymes such as cathepsin K to degrade the surrounding matrix, making calcium and phosphate available for nearby osteoblasts to incorporate into newly formed bone [12, 13]. This extracellular acidification, where the local pH drops to ∼4.5, facilitates mineral dissolution and releases calcium and phosphate ions essential for bone remodelling [14]. This process highlights the essential role of osteoclasts in converting βTCP into a biologically accessible form of calcium. However, the efficiency and consistency of this resorption process may vary depending on the *in vivo* environment, particularly in segmental bone defects where vascularization, cellular recruitment and mechanical stability are often compromised. These factors introduce uncertainty regarding the long-term performance of βTCP in large or anatomically complex defects.

The cellular component of BTE is also critical, with mesenchymal stem cells (MSCs) being extensively studied for their potential to differentiate into osteogenic lineages. While bone marrow-derived MSCs (BMSCs) have been a traditional choice, their clinical application is hindered by invasive harvesting procedures and low yields [15]. In contrast, multipotent stromal adipose-derived stem cells (ADSCs) offer a less invasive alternative, providing higher cell yields and demonstrating robust osteogenic potential [16, 17]. Furthermore, ADSCs exhibit faster proliferation rates, making them attractive for large-scale applications [18]. However, autologous ADSCs still carry some donor-site morbidity, prompting interest in allogeneic ADSCs. These allogeneic cells offer an off-the-shelf solution, eliminating harvesting procedures and broadening accessibility. Despite their promise, questions remain about the immunogenicity, long-term integration and bone regenerative efficacy of allogeneic ADSCs compared to autologous cells, particularly *in vivo* [5]. Importantly, MSCs, including ADSCs, do not act solely as osteoblast precursors but also influence osteoclast activity through paracrine signalling. They secrete regulatory factors such as receptor activator of nuclear factor kappa-B ligand (RANKL), osteoprotegerin (OPG) and macrophage colony-stimulating factor (M-CSF), thereby modulating osteoclast differentiation and function within the bone microenvironment [19, 20]. This crosstalk ensures that bone formation and resorption remain coupled, highlighting that the regenerative potential of ADSCs depends not only on their osteogenic differentiation but also on their capacity to orchestrate balanced osteoblast–osteoclast dynamics.

Here, we describe an *in vivo* scapular bioreactor model to address the aforementioned challenges. This model was specifically designed to compare ectopic bone formation associated with various osteoinductive and osteoconductive candidates. Importantly, it allowed simultaneous interaction with bone and periosteum, providing a dynamic environment for bone regeneration. The model’s ability to yield sufficient sample sizes ensured robust statistical analyses while minimizing animal use. This study was designed to systematically evaluate the influence of bioresorbable scaffold architecture on osteogenesis, with particular emphasis on the role of pore size, the inclusion of osteogenically differentiated ADSCs as a cellular component, and the comparison between autologous and allogeneic ADSC sources. By assessing how these factors interact to promote new bone formation, the research further aims to address key parameters essential for successful BTE strategies.

## Materials and methods

### Design and additive manufacturing of the polyetherketone bioreactors

**Figure 1:**
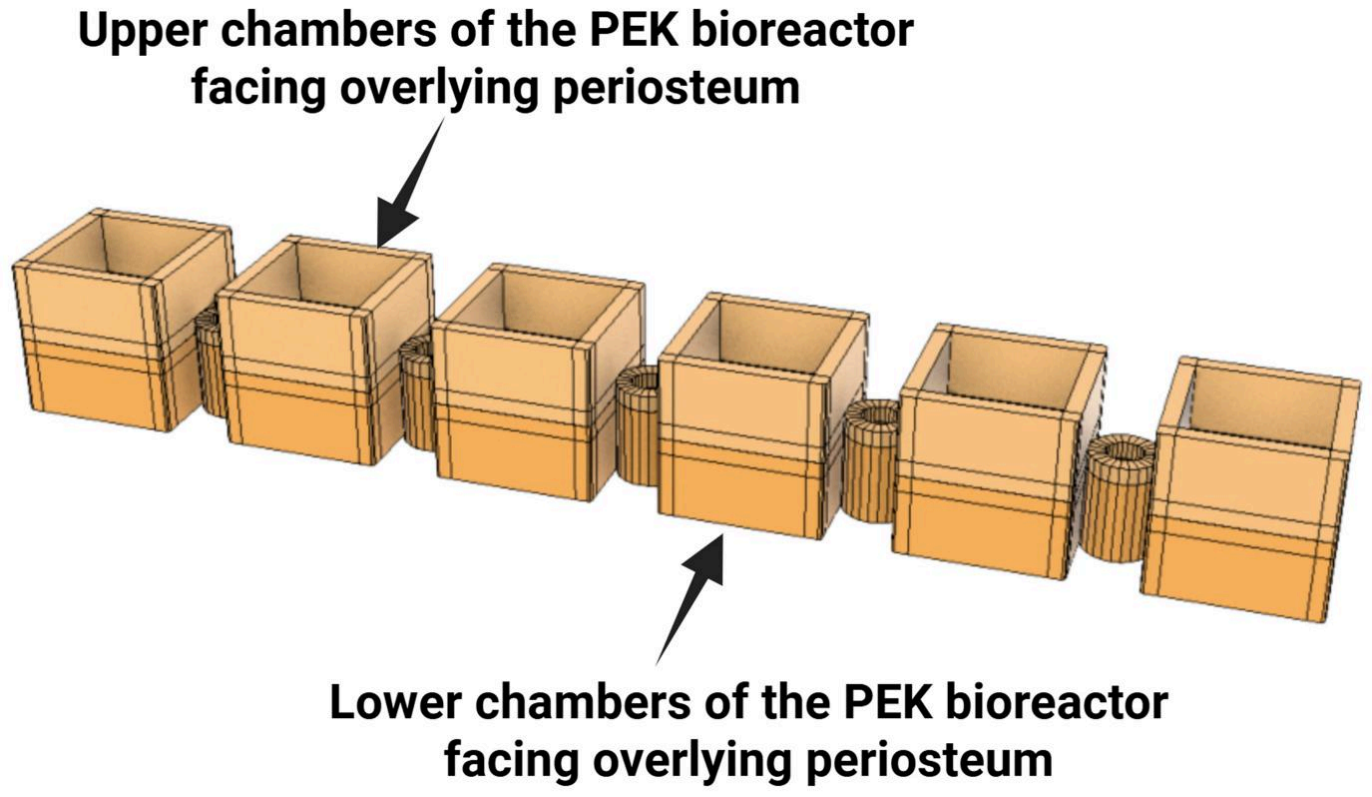
Computerized design of PEK bioreactor chambers. Each chamber has one open face, either contacting the overlying scapular periosteum (upper chamber) or contacting the underlying scapular bone (lower chamber).

The custom-designed bioreactor chambers were developed to provide a controlled *in vivo* environment for evaluating scaffold-guided bone regeneration under two distinct anatomical conditions: one chamber facing the overlying periosteum (upper chamber) and the other chamber facing the underlying native bone (lower chamber) (Figure 1). This configuration allowed simultaneous assessment of bone formation in different biological niches within the same animal model, ensuring the application of the replacement, reduction and refinement principle in conducting animal studies. The bioreactor strip consisted of six individual chambers, each volume measuring 8×8 mm in length and 5 mm in depth and a 1 mm-thick wall enclosed each bioreactor, leaving one open surface. Screw rings were incorporated to secure the bioreactors to the bone. The bioreactors were designed using Autodesk 3ds Max 2022 (Autodesk, San Rafael, USA) with polygonal modelling and the extrude modifier. To achieve smooth surfaces and eliminate sharp edges during printing and post-processing, smoothing techniques like chamfering and edge filleting were applied during the 3D modelling process. In addition, identification markers were added using the Boolean union modifier. The finalized designs were exported as Stereolithography (STL) files and manufactured through laser sintering (LS) on an EOS EOSINT P 800 (EOS Group, Hamburg, Germany) with polyetherketone (PEK) material, a ketone-based, semi-crystalline thermoplastic. After printing and cooldown, the scaffolds were removed from the powder cake of the finished print and blasted free of unsintered powder with dry ice using a ColdJet Microclean at 1.8 bar gauge pressure and a feed rate of 0.15 kg/min (ColdJet, USA).

### Design of the selectively polymerized β-tricalcium phosphate porous scaffold

**Figure 2:**
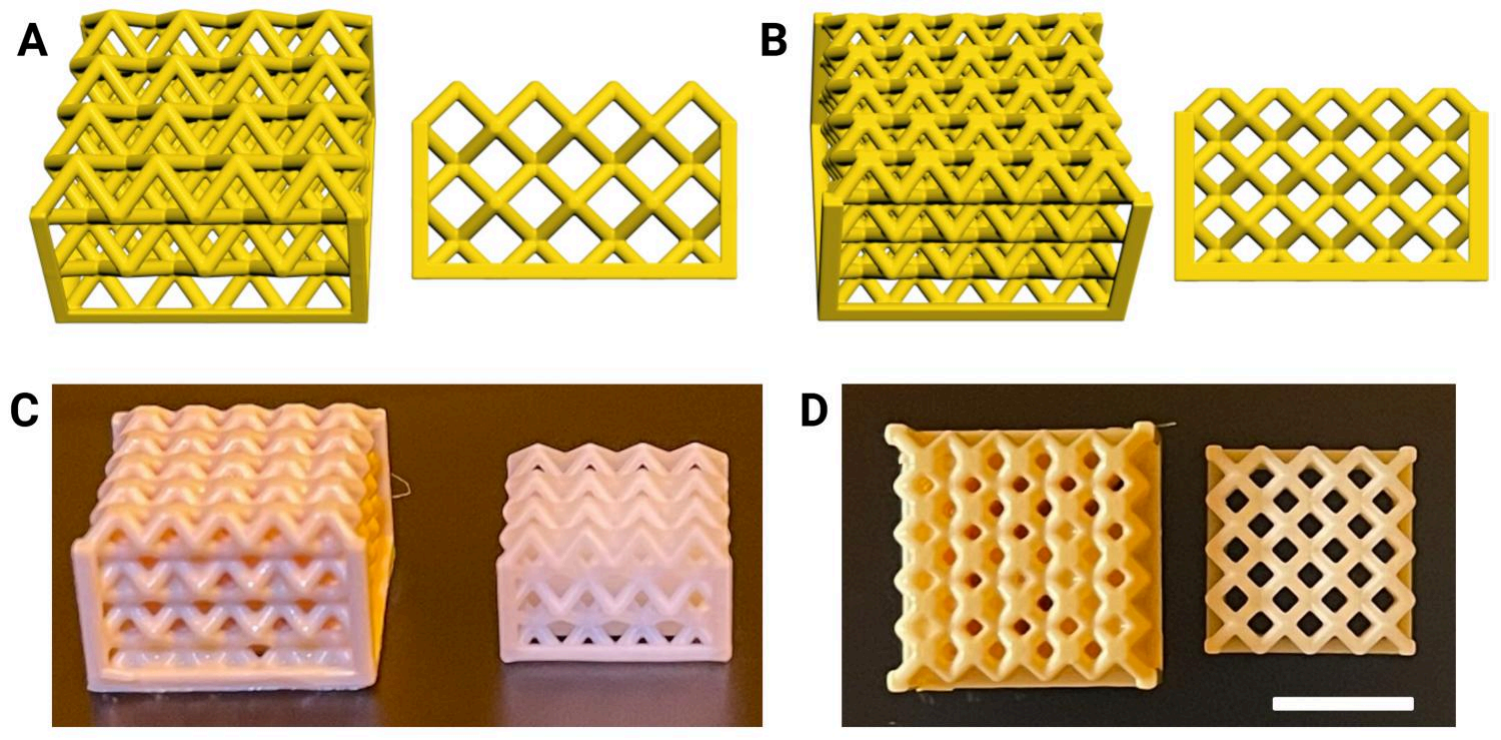
Computer-aided design (CAD) models and additive manufacturing of low- and high-density spherical porous βTCP. (**A** and **B**) Both scaffold types feature uniform strut diameters of 0.4 mm. The low-density scaffold design incorporates a larger unit cell with a repeat interval of 1.875 mm with pore size of 0.93 mm, whereas the high-density design employs a more compact unit cell repeating at 1.5 mm intervals, resulting in increased structural density with each pore measuring 0.66 mm. (**C** and **D**) Additively manufactured βTCP scaffolds of both types. Scale bar: 5 mm.

The βTCP scaffolds were designed using computer-aided design (CAD) software equipped with tessellation functionality, enabling the creation of ‘unit cell’ structures within a defined volume. Differentiated scaffold densities were developed to investigate the effects of porosity on post-operative outcomes. Two variants, one high density and one low density, were created, facilitating comparative analysis of scaffold performance. Both incubator implants employed the same face-centred cubic unit cell structure, which was also used in the primary mandible implant. To achieve the different densities, cell size and tessellation frequency were modified, resulting in distinct high- and low-density structures. Each implant was designed to fit within an 8×8×5 mm volume (equivalent to the bioreactor chamber), with tolerancing gaps integrated into the design files to ensure wall clearance. This design refinement yielded an effective implant bounding volume of 7.8×7.8×4.9 mm. To enhance both structural integrity and 3D printability, a 0.4 mm beam was added around the base and vertical edges of each scaffold. This reinforcement was incorporated without affecting the overall dimensions of the implant. The Large Pore (high porosity) scaffold (Figure 2A) featured a 1.875 mm unit cell tessellated throughout the structure while maintaining a 0.4 mm beam size, resulting in a more porous scaffold with each pore measuring 0.93 mm. In contrast, the Small Pore (low porosity) implant (Figure 2B) used the same 0.4 mm beam size but with a smaller 1.5 mm unit cell, producing a denser scaffold architecture with each pore measuring 0.66 mm.

### Additive manufacturing of selectively polymerized βTCP porous scaffold

The manufacturing process was conducted on a Lithoz Cerafab 7500 ceramic 3D printer (Lithoz GmbH, Austria) using LithaBone TCP300 material (Lithoz GmbH, Austria). Scaffolds were printed over several runs, with post-processing of each batch, involving removal of residual slurry with compressed air and a cleaning solution, LithaSol 30 (Lithoz GmbH, Austria), applied via an airbrush tool, followed by sintering. Sintering was performed over 96 h, following a gradual temperature increase up to 1200°C. This thermal process facilitated polymer removal and crystallization of the remaining ceramic material. Upon completion, the sintered implants underwent inspection and were subsequently transferred to designated containers until further use (Figure 2C and D).

### Plasma immersion ion implantation treatment of PEK bioreactors

Plasma immersion ion implantation (PIII) treatments were used to activate the surface of the PEK bioreactors prior to *in vivo* implantation to induce hydrophilic wetting properties and create opportunities for the covalent binding of biomolecules, as well as to increase the cleaning efficiency of the bioreactor prior to ultrasonication cleaning [21]. The PIII treatment was performed using a large volume system operating according to the protocol described in detail previously [22]. Briefly, for the main PIII treatment, the bioreactors were immersed in pure nitrogen gas at various pressures to ensure penetration of the plasma into the insides of the bioreactors. The plasma discharge was excited with 10 kV negative pulses at the frequency of 1000 Hz. The total treatment time was 40 min. The operating principles of the dielectric barrier discharge customized for medical applications of PIII have been described in detail in prior work [23]. All bioreactors were steam sterilized and sealed in sterile pouches until further use.

### Harvesting, expansion and encapsulation of ADSCs in gelatine methacryloyl

Approximately 50 g of adipose tissue was harvested from the back of anesthetized sheep using a scalpel, placed in phosphate-buffered saline (PBS) containing 5% penicillin/streptomycin (P/S; Thermofisher Scientific, Australia) and maintained cold (4°C) on wet ice during transport. Under sterile conditions in a Biological Safety Cabinet, the collected tissue was washed several times with PBS containing 5% P/S, drained and transferred to a sterile tissue culture dish and minced finely using a scalpel blade. The resultant minced tissue was weighed and transferred to a tube with equal (w/v) 0.075% collagenase IV (Sigma) in Dulbecco’s modified Eagle medium (DMEM, Thermofisher Scientific, Australia) containing 2% P/S. The tissue was incubated for 2 h at 37°C with gentle agitation every 5 min and pipetting up and down several times every 30 min using a 25 mL serological pipette. After digestion, an equal volume of 20% foetal bovine serum (FBS; Thermofisher Scientific, Australia) and 2% P/S in DMEM were added to neutralize enzyme activity, followed by centrifugation at 760 × *g* for 10 min. The fat layer was removed from the tube and the collagenase solution aspirated, followed by resuspension of the pellet in an equal volume of DMEM containing 1%P/S. Samples were again centrifuged at 760 × *g* for 10 min and the supernatant aspirated without disturbing the cell pellet. The pellet was resuspended in DMEM with 10% FBS and 1% P/S and filtered using a 100 µm nylon cell strainer. Cells were counted and seeded at a minimum cell density of 10×10^3^ cells per cm^2^ in tissue culture flasks in DMEM with 10% FBS, 1% P/S and 10 ng/mL fibroblast growth factor 2 (bFGF; Thermofisher Scientific, Australia). Nonadherent cells were removed after 72–96 h culture, with medium changed every 3–4 days.

### Assembly of SP-βTCP and PIII-treated polyetherketone bioreactors

β-TCP scaffolds were dry heat sterilized in sealed sterilization pouches (double bagged) at 170°C for 1 h, with a gradual ramp up and ramp down at 10°C per 10 min to minimize warping of the scaffolds. Following sterilization, β-TCP scaffolds were then placed within plasma-treated polyetherketone (P-PEK) bioreactors under sterile conditions within a biosafety cabinet.

### Encapsulation of ADSCs-laden gelatine methacryloyl within P-PEK bioreactors

ADSCs suspended in gelatine methacryloyl (GelMA) were selectively incorporated into the Large Pore SP-βTCP scaffolds to ensure efficient hydrogel infiltration and uniform cell distribution throughout the construct. Due to the restricted pore architecture of the Small Pore scaffolds, which limits homogeneous hydrogel penetration, these scaffolds were implanted without cellular or hydrogel infill and served as non-infused controls for comparison.

Following expansion of ADSCs in tissue culture flasks, once cells reached 90% confluence, adherent cells were harvested by trypsinization (0.25% trypsin-EDTA, Thermofisher Scientific, Australia) and 6–10×10^6^ ADSCs encapsulated per ml of GelMA (TRICEP, Australia) at a final concentration of 5%, with 0.25% lithium phenyl-2,4,6-trimethylbenzoylphosphinate. GelMA solution (derived from porcine skin gelatine, type A, 300 bloom, 37% ± 4% degree of functionalization) was prepared as previously described [24]. Implants with autologous ADSCs contained ADSCs from the same sheep being implanted, whereas implants with allogeneic ADSCs were derived from an equal mix of ADSCs derived from two or more sheep, which were not the sheep that would be implanted. Autologous ADSCs-laden GelMA (AUTG) or allogeneic ADSCs-laden GelMA (ALTG) was infused into SP-βTCP scaffolds within each P-PEK bioreactor using a syringe with an 18 G needle. Crosslinking was performed after infusing using a 405 nm photocuring toolhead of a CELLINK BIOX6 bioprinter (CELLINK, Sweden). UV was delivered from a height of 1 cm above the scaffold for 120 s. Crosslinked constructs were then transferred under sterile conditions to 2 cm deep, 10 cm diameter tissue culture dishes and cultured in 10% FBS, 1% P/S and 10 ng/mL bFGF in DMEM for 5 days at 37°C in a 5% CO_2_ humidified incubator for further cell expansion *in situ*.

### Osteogenic differentiation of encapsulated ADSCs

To pre-differentiate ADSCs within constructs to osteogenic lineage, culture media was replaced with osteogenic pre-differentiation media consisting of DMEM supplemented with 10% FBS (Thermofisher Scientific, Australia), 1% P/S (Thermofisher Scientific, Australia), 50 µM L-ascorbic acid 2-phosphate sesquimagnesium salt (Sigma, Australia), 10 mM β-glycerol phosphate (Sigma, Australia) and 100 nM dexamethasone (Sigma, Australia). ADSCs-laden GelMA within SP-βTCP constructs were cultured at 37°C in a 5% CO_2_ humidified incubator for an additional 7 days, with a half medium change every 3–4 days, before transplantation to sheep.

### Implantation surgery

**Figure 3:**
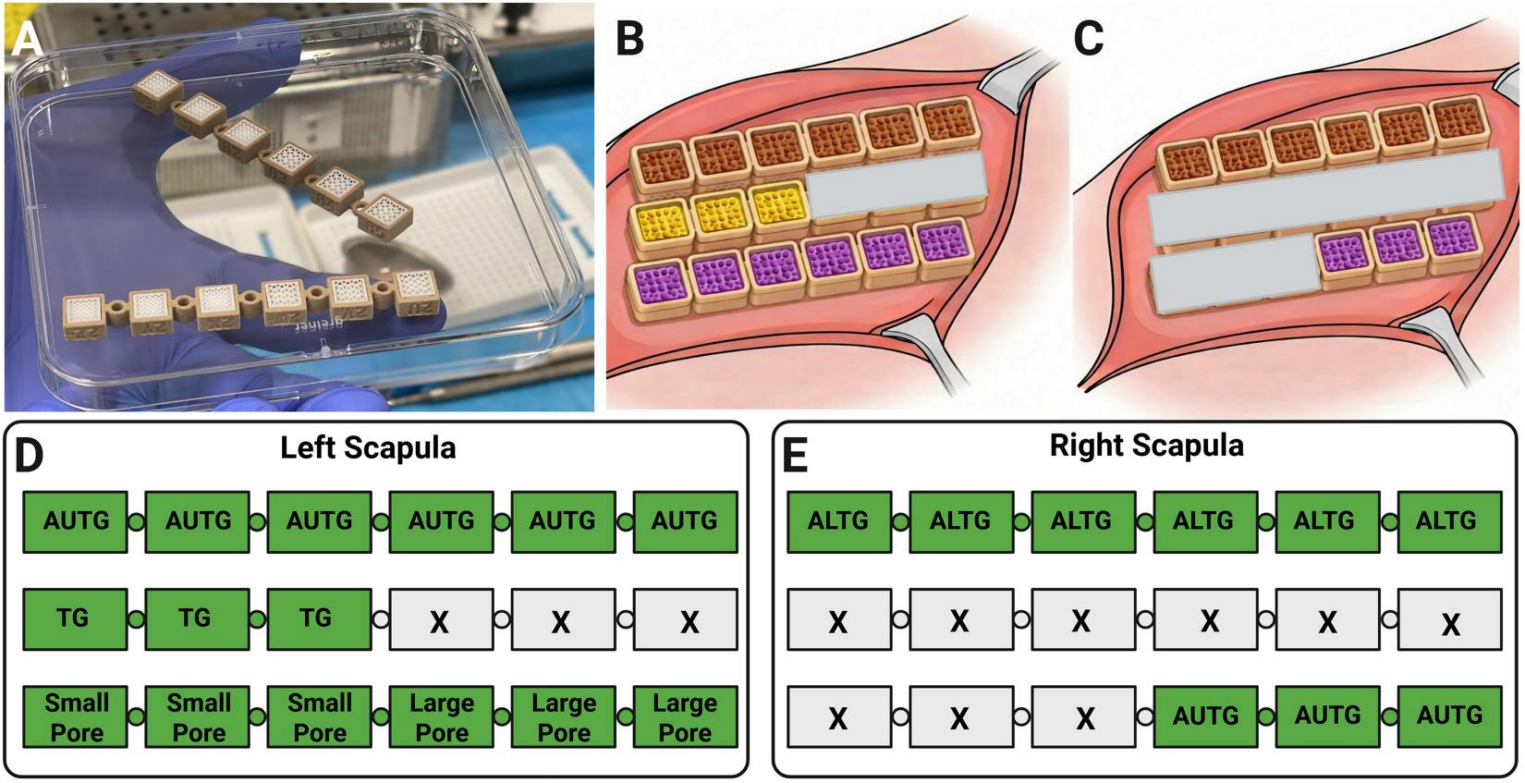
Preparation of P-PEK bioreactors with SP-βTCP porous scaffolds and surgical implantation in an ovine model. (**A**) P-PEK bioreactor chambers pre-loaded with SP-βTCP scaffolds prior to implantation were maintained under sterile conditions. (**B**) Experimental configurations included SP-βTCP scaffolds infused with GelMA-ADSC or GelMA alone, as well as small pore and large pore SP-βTCP scaffolds without any infiltration. (**C**) Intraoperative placement of P-PEK bioreactors onto the scapular bone surface, with chambers secured in position for subsequent *in vivo* evaluation of bone formation. Grey areas in (**E**) and (**F**) denote bioreactors unrelated to this study. (**D** and **E**) Schematic demonstrating the placement of bioreactors containing different groups in both left and right scapula. Bioreactors labelled as ‘X’ were unrelated to this study.

The study was approved by the animal ethics committee of the University of Sydney (ethics approval number: 2022/2232). Six female sheep, aged 7–8 years and weighing between 70 and 80 kg, were housed in groups on straw bedding and maintained on a standard diet of chaff and hay for a minimum of 2 weeks prior to the surgery. All animals were deemed healthy based on a physical examination before the procedure. Premedication included 0.3 mg/kg of methadone (Methodyne, Jurox, Australia) and 0.2 mg/kg of midazolam (Ilium diazepam, Troy Animal Healthcare, Australia), administered through a pre-inserted intravenous (IV) cannula. General anaesthesia was induced using IV propofol (Propofol-Lipuro 1%, B. Braun Melsungen AG, Germany; 2–4 mg/kg). Anaesthesia was maintained with isoflurane (Isothesia NXT, Piramal Pharma Limited, India; end-tidal concentration 1.4%–1.8%) delivered in an air–oxygen mixture. Sheep were mechanically ventilated to maintain normocapnia. Sheep received prophylactic antibiotics prior to surgical incision and multimodal analgesia according to the LAS Sheep Anaesthesia & Analgesia SOP, Laboratory Animal Services, University of Sydney. All bioreactors, pre‑loaded with β‑TCP scaffolds and prepared with either ADSCs plus GelMA, GelMA alone or no cellular component, were maintained under sterile conditions until surgery (Figure 3A). The surgical site, over the left scapular infraspinous muscle, was shaved and sterilized with chlorhexidine and povidone iodine. A 20 cm incision was made over the infraspinatus and teres major muscles, which were carefully detached from the scapula, lifting the periosteum with the muscle using a periosteal elevator to ensure the periosteum and vascular supply remained undamaged.

The experimental groups were organized into two sets of 18 bioreactors, one set for the left scapula and one set for the right scapula (however, there were a total of 12 sets of bioreactors containing groups unrelated to this study in both left and right scapula, marked as grey in Figure 3D–E), performed 4 weeks later, allowing the sheep to remain ambulant through the entire experiment. During surgical implantation, the scapula bone was exposed by detaching the infraspinatus muscle and the underlying periosteum from the scapular bone. The P-PEK bioreactor chambers were organized into three rows of six bioreactors, organized superior, middle and inferior on each scapula. While the left scapula accommodated the following groups of bioreactors: (i) SP-βTCP scaffold with small-sized pores (Small Pore; without cells or GelMA, *n* = 3), (ii) SP-βTCP scaffolds with large-sized pores (Large Pore; without cells or GelMA, *n* = 3), (iii) SP-βTCP scaffolds with large-sized pores containing GelMA hydrogel (TG; cultured in osteogenic medium for 21 days, *n* = 3), (iv) SP-βTCP scaffolds with large-sized pores containing autologous ADSCs and GelMA (AUTG; cultured in osteogenic medium for 21 days, *n* = 6), the following groups of bioreactors were implanted in the right scapula: (i) SP-βTCP scaffold with large-sized pores containing allogeneic ADSCs and GelMA (ALTG; cultured in osteogenic medium for 21 days, *n* = 6) and (ii) AUTG (cultured in osteogenic medium for 21 days, *n* = 3) (Figure 3B and C). Both layers of bioreactors were secured to the scapular bone using titanium screws. The subcutaneous fascia and muscle layers were closed with interrupted 3-0 Vicryl (Ethicon, USA) sutures, while the skin was closed using a running subcuticular 3-0 monofilament Poliglecaprone 25 suture (Monocryl, Johnson & Johnson, Australia).

Sheep were monitored throughout the study for any adverse local or systemic immune reactions. Unfortunately, two mortalities occurred unrelated to the experimental procedure: one sheep aspirated during the surgery and was euthanized on the same day, while the other sheep died from an undetermined cause. After 16 weeks of implantation (12 weeks post-surgery right scapular surgery), the sheep were sedated and euthanized according to the standard operation procedure for Euthanasia and Humane Killing of Pigs and Sheep from Laboratory Animal Services at Charles Perkins Centre, The University of Sydney. All the bioreactors from both the right and left scapula were harvested, and the bioreactor samples were preserved in 10% neutral buffered formalin for subsequent tissue fixation.

### Micro-computed tomography scanning and analysis of the harvested P-PEK bioreactor contents

The micro-computed tomography (µCT) procedure was conducted in a PC2 laboratory environment to ensure compliance with biosafety regulations. Specimens were wrapped with Parafilm M (Amcor plc, Switzerland) to maintain integrity during scanning. A wrapped specimen was then securely placed on the scanning bed of the MILabs µ-CT system (MILabs, The Netherlands). Using X-ray preview as the imaging source, CT positions were adjusted to ensure the specimen was centrally aligned within the scan field. Scanning was performed using the following parameters: Magnification set to Ultrafocus, Step Angle at 0.75°, 16 Projections per Step, an estimated dose of 752 mGy, Tube Voltage at 55 kV, Tube Current at 0.19 mA and Exposure Time of 20 ms per projection. Each scan took ∼8 min and 38 s to complete. The acquired scanning data were reconstructed using the MILabs Acquisition software CT (MILabs, The Netherlands) on a DELL PowerEdge workstation (Round Rock, USA) equipped with a 64-bit operating system, an Intel Xeon CPU @ 2.6 GHz, and 128 GB of RAM. The reconstructed imaging files were subsequently exported at 20 µm resolution in the Neuroimaging Informatics Technology Initiative (.NIfTI) format for further analysis.

Reconstructed .NIfTI µCT image files for each upper and lower bioreactor chamber from both control and experimental groups were analysed using Imalytics Preclinical 3.0 software (v3.0.1.3; Gremse-IT GmbH, Germany) following a customized six-step protocol. First, individual bioreactor chambers were isolated using Imalytics clipping tools to define regions of interest (ROIs), ensuring inclusion of only one chamber per ROI by carefully viewing axial, sagittal and coronal planes to exclude surrounding structures. Next, segmentation was performed using voxel brightness thresholding to isolate SP-βTCP scaffold regions, with values manually selected for each chamber to exclude new bone. New bone formation was then identified separately using a fixed minimum threshold of 1400 and an upper threshold equivalent to the scaffold threshold minimum. The segmented data were subsequently analysed using the Bone Statistics plugin to quantify both bone volume (BV) and scaffold volume. To validate threshold accuracy, scaffold volumes were compared to two reference values: the original CAD model volumes and volumes from pre-implantation µCT scans processed using the same protocol. Additionally, a lower boundary was established using ten visibly degraded scaffolds identified by visual inspection. Final scaffold volume values were expected to fall between these upper and lower limits; if not, threshold parameters were revisited and adjusted accordingly. This standardized thresholding protocol was consistently employed for analysing new bone formation in both the upper and lower chambers, allowing for accurate and reproducible results across experimental groups.

### Histology

Bioreactor chambers were selected for histological processing based on regions of interest identified through µCT analysis. After removing the surrounding soft tissue using a precision band saw, each sample was bisected and prepared for resin embedding. The selected specimens were dehydrated through a graded series of ethanol solutions and subsequently infiltrated and embedded in Technovit 9100 methyl methacrylate resin without prior decalcification. Embedded blocks were trimmed to dimensions suitable for standard 75 × 25 mm microscope slides and sectioned to ∼50 µm thickness using the EXAKT cutting and grinding system (EXAKT Advanced Technologies GmbH, Germany). The resulting ground sections were stained following a Goldner’s trichrome protocol as reported previously [25, 26], which included sequential application of Weigert’s haematoxylin, acid Fuchsin-Ponceau, tungstophosphoric acid-orange G and light green, interspersed with acetic acid rinses. Following staining, slides were air-dried, cleared in xylene and coverslipped. Imaging was conducted at 5× magnification using a Zeiss Axio Observer 7 microscope (Carl Zeiss Microscopy, Germany) operated in tile scan mode to capture full-section images.

To complement the µCT-based verification of new bone formation, a semi-quantitative histological image analysis was performed on selected Goldner’s trichrome-stained sections using ImageJ (National Institutes of Health, USA). Only those histological sections that had been spatially matched to the corresponding µCT verification slices were included in this analysis, allowing direct assessment of the bone regions identified during the validation process.

For each matched histological section, the newly formed bone area (BA) was manually delineated based on morphological appearance and staining characteristics consistent with mineralized bone tissue. The total available tissue area (TA) within the scaffold chamber was then defined for each section. From these measurements, two parameters were calculated: bone area (BA, mm^2^) and the bone area-to-total area ratio (BA/TA%). These measurements were performed across the experimental groups included in this study.

As this histological image analysis was performed only on the subset of sections used for µCT–histology verification, the sample size was limited and not powered for formal statistical comparison. Therefore, the data are presented descriptively to support interpretation of the validation findings rather than as a standalone quantitative endpoint. The corresponding results of the histological image analysis are added to the Supplementary Data section.

### Statistical analysis

Quantitative data on new bone volumes were subjected to statistical analysis using GraphPad Prism (version 10.4.1, GraphPad Software, Inc.). A two-way analysis of variance (ANOVA) with Tukey’s multiple comparison test was performed to compare differences among the experimental groups. *P<*0.05 was considered statistically significant for all analyses.

## Results

### Clinical monitoring and inflammatory response assessment

Throughout the postoperative and follow-up periods, all sheep remained clinically stable with no observable signs of local or systemic inflammation. Daily monitoring revealed no swelling, erythema or abnormal tissue reactions at the implantation sites, and body temperature and behaviour remained within normal ranges. Wound healing progressed without complications, and no indications of graft rejection were detected. These findings persisted after discontinuation of the short postoperative course of anti-inflammatory analgesics administered for surgical pain management. Collectively, the clinical observations indicated an absence of detectable inflammatory responses associated with the implanted constructs.

### µCT analysis of new bone formation

**Figure 4:**
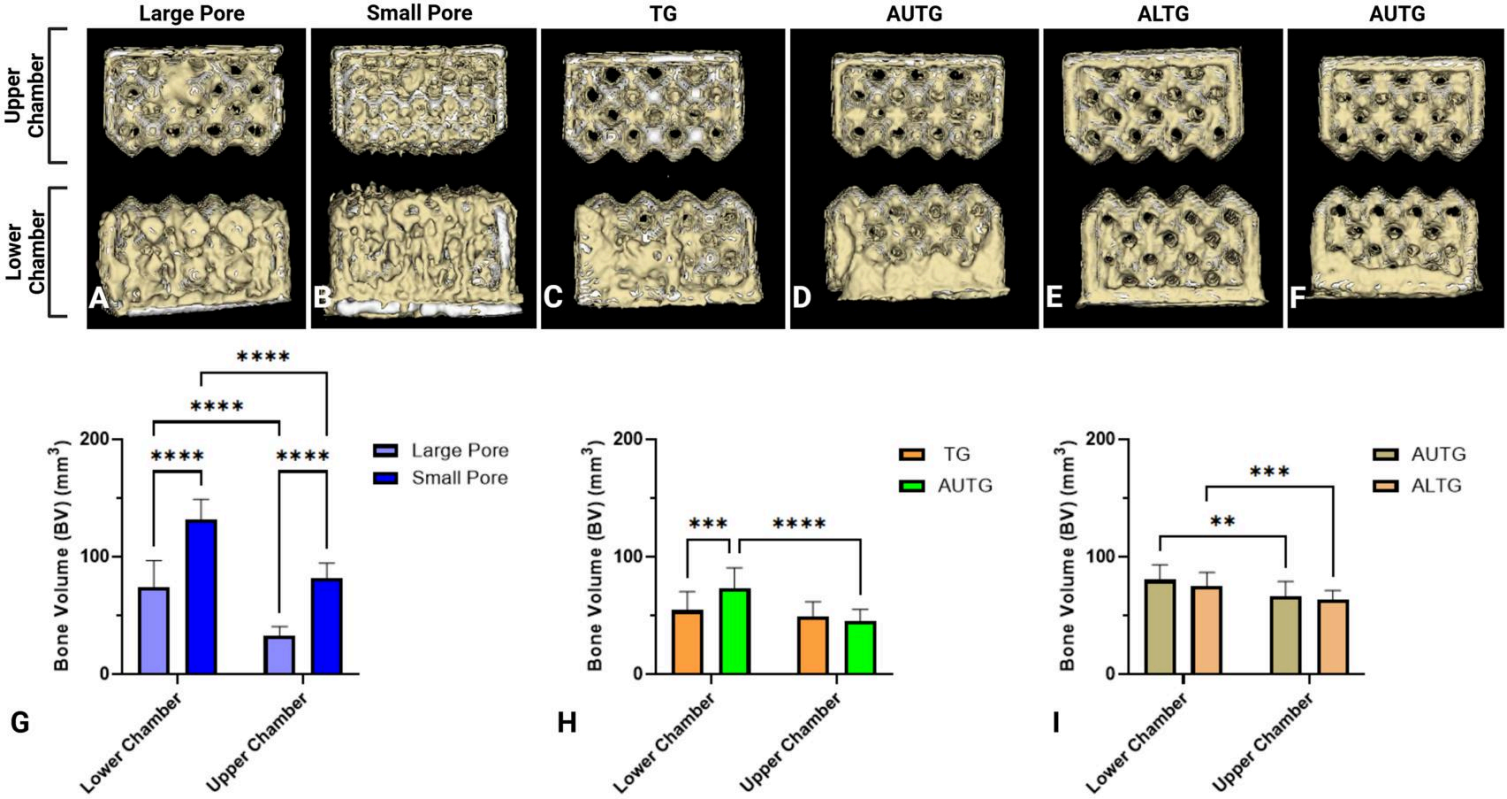
Quantitative analysis of bone volume (BV) within upper and lower bioreactor chambers across different scaffold configurations and experimental groups. (**A–F**) Representative μCT images showing new bone formation within SP-βTCP scaffolds from different experimental groups: (**A**) Large P, (**B**) Small Pore, (**C**) TG, (**D**) AUTG, (**E**) ALTG and (**F**) AUTG from both upper and lower chambers, illustrating variations in bone regeneration patterns within upper and lower bioreactor chambers. Gold colour represents new bone formed around the SP-βTCP scaffold and white colour represents the SP-βTCP scaffolds. **(G–I**) Bar graphs showing BV analysis in upper vs lower chambers for each scaffold type: Large Pore vs Small Pore (**G**), TG vs AUTG (**H**) and AUTG vs ALTG groups (**I**). Statistical significance: **P < 0.01, ***P < 0.001, ****P < 0.0001. Data are presented as mean ± SD.

µCT was used to compare new bone formation in the upper and lower bioreactors after 12 and 16 weeks of implantation. Firstly, the Small Pore group exhibited higher BV at 16 weeks compared to the Large Pore group in both lower (*P* < 0.001; *F* (1, 44) = 133.3) and upper (*P* < 0.001; *F* (1, 44) = 96.68) bioreactors, suggesting that smaller pores within the SP-βTCP scaffolds may enhance bone regeneration (Figure 4A). Similar results were observed in estimating the bone volume to total volume ratio (BV/TV%) in both lower (*P* < 0.001; *F* (1, 44) = 133.3) and upper bioreactors (*P* < 0.001; *F* (1, 44) = 96.68) (Figure S1A).

When comparing the TG and AUTG groups, the AUTG group showed greater bone volume in the lower bioreactors after 16 weeks (*P* = 0.0016, *F* (1, 68) = 4.496; Figure 4B), but no difference was observed between groups in the upper bioreactors (*P* = 0.8375, *F* (1, 68) = 21.71). When comparing the AUTG and ALTG groups, there was no difference in bone volume in either the upper (*P* = 0.871, *F* (1, 68) = 2.35) or lower bioreactors (*P* = 0.498, *F* (1, 68) = 22.07) at 12 weeks (Figure 4C). This indicates that the ADSCs may be advantageous in bone regeneration, but that the origin of the ADSCs is less important.

When comparing the TG and AUTG groups, as well as the AUTG and ALTG groups, the BV/TV% values were found to be similar in both lower and upper bioreactors (Figure S1B and C).

Remarkably, BV was consistently greater in the lower bioreactors compared to the upper bioreactors for AUTG and ALTG groups (*P* = 0.012 and *P* = 0.002, respectively), suggesting that contact with native bone was more conducive to osteogenesis than contact with periosteum.

### Histological verification of µCT analysis

**Figure 5:**
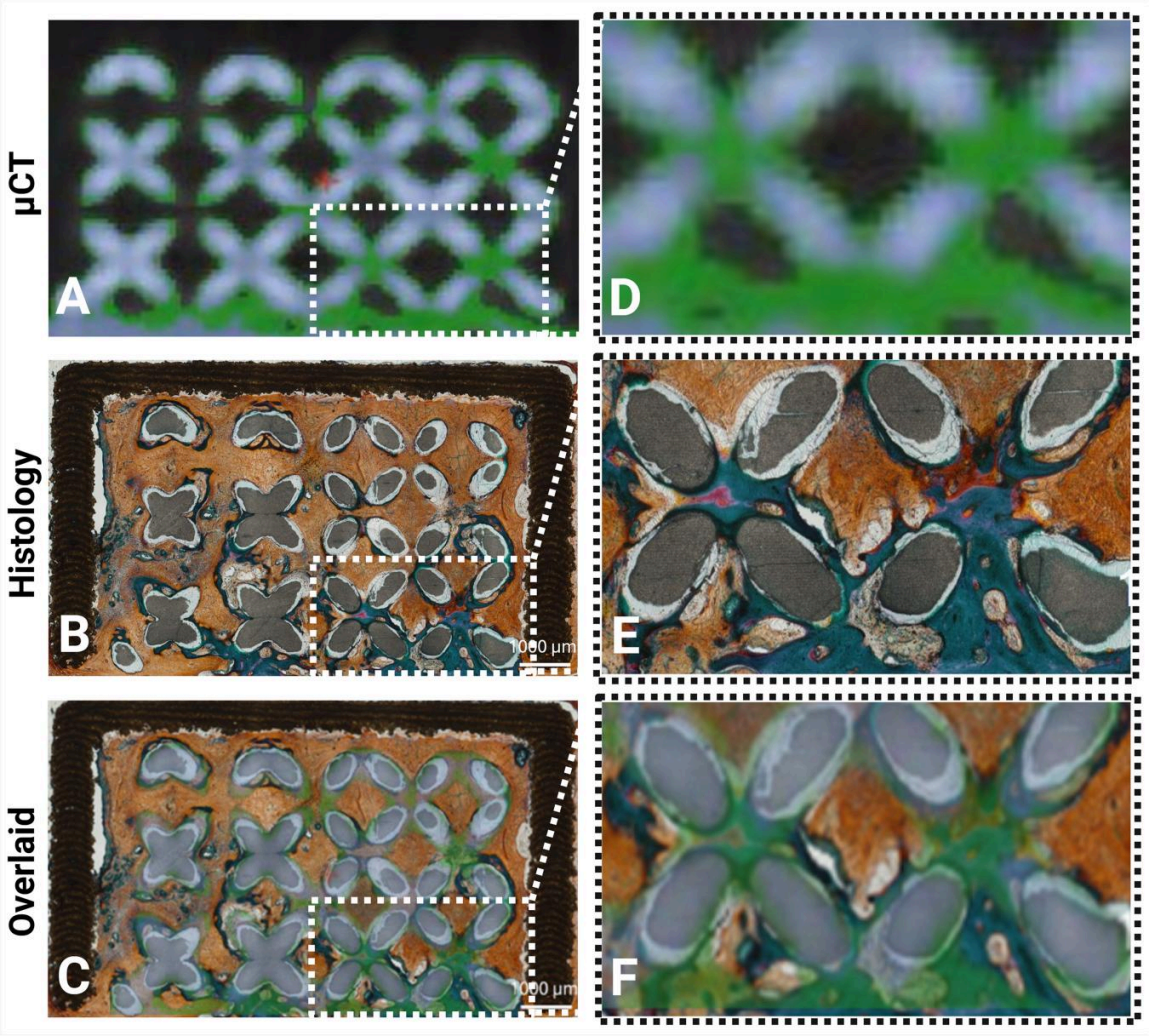
AUTG Bioreactor: μCT–histology comparison for mineralized tissue assessment. (**A**) μCT reconstruction of the AUTG bioreactor chamber showing segmented mineralized tissue (green) within the β-TCP scaffold. (**B**) Corresponding Goldner’s trichrome-stained histological section of the same slice, with mineralized bone in blue, osteoid in red–orange and scaffold material in grey. (**C**) Overlay of μCT and histology images illustrating spatial correspondence between modalities. (**D–F**) Magnified views of the highlighted region in **A–C**, respectively, showing fine-scale alignment of scaffold architecture and mineralized tissue distribution. Scale bars: 1000 μm.

**Figure 6:**
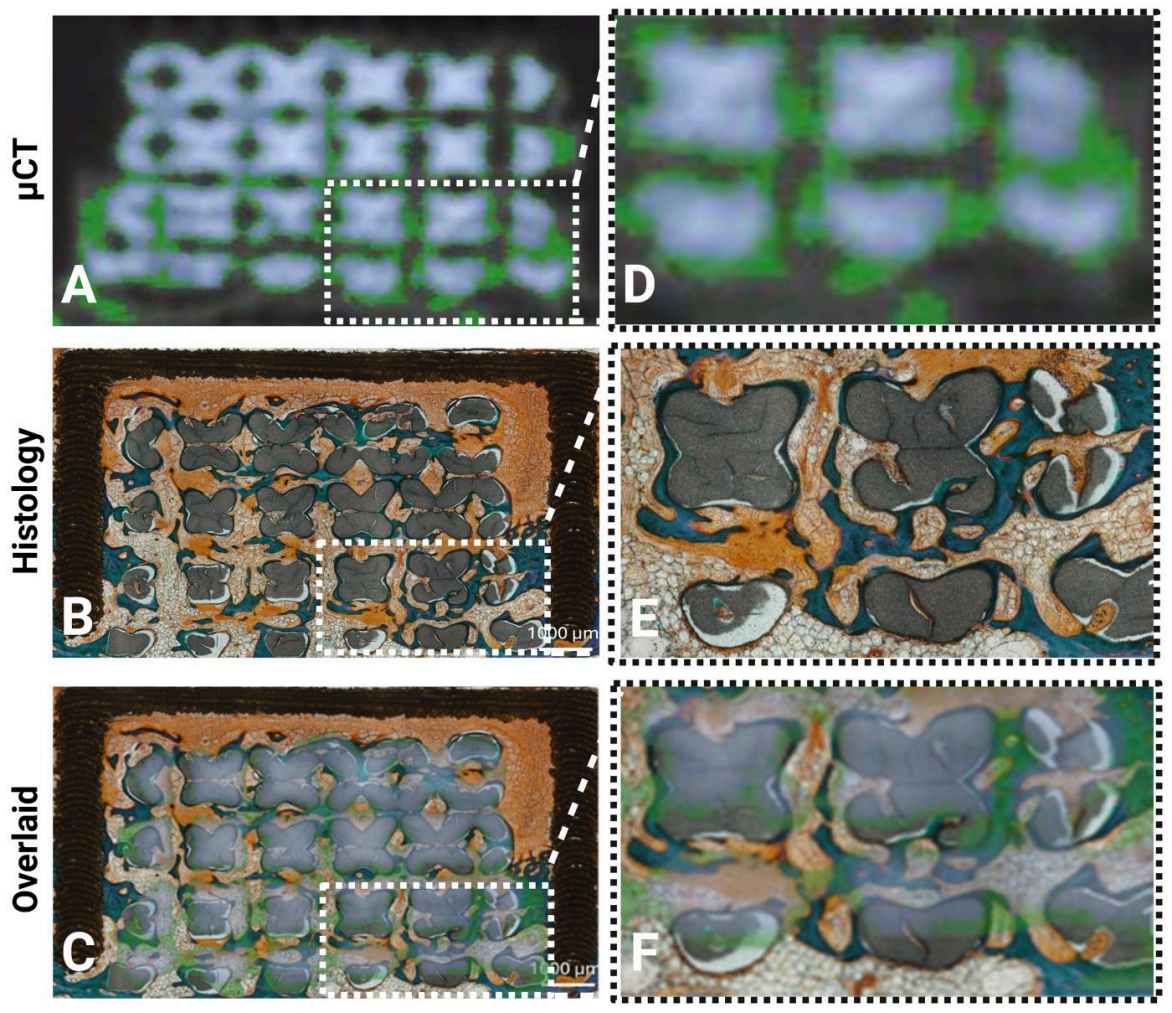
Small Pore bioreactor (lower chamber): μCT–histology comparison for mineralized tissue assessment. (**A**) μCT reconstruction of the Small Pore scaffold with segmented mineralized tissue (green) distributed along struts and within pore spaces. (**B**) Goldner’s trichrome-stained histological section of the same slice, showing mineralized bone (blue), osteoid (red–orange) and scaffold (grey). (**C**) Overlay of μCT and histology images demonstrating alignment of scaffold geometry and mineralized regions. (**D–F**) Enlarged views of the boxed region in **A–C**, highlighting detailed correspondence between segmented μCT mineralization and histological bone staining. Scale bars: 1000 μm.

**Figure 7:**
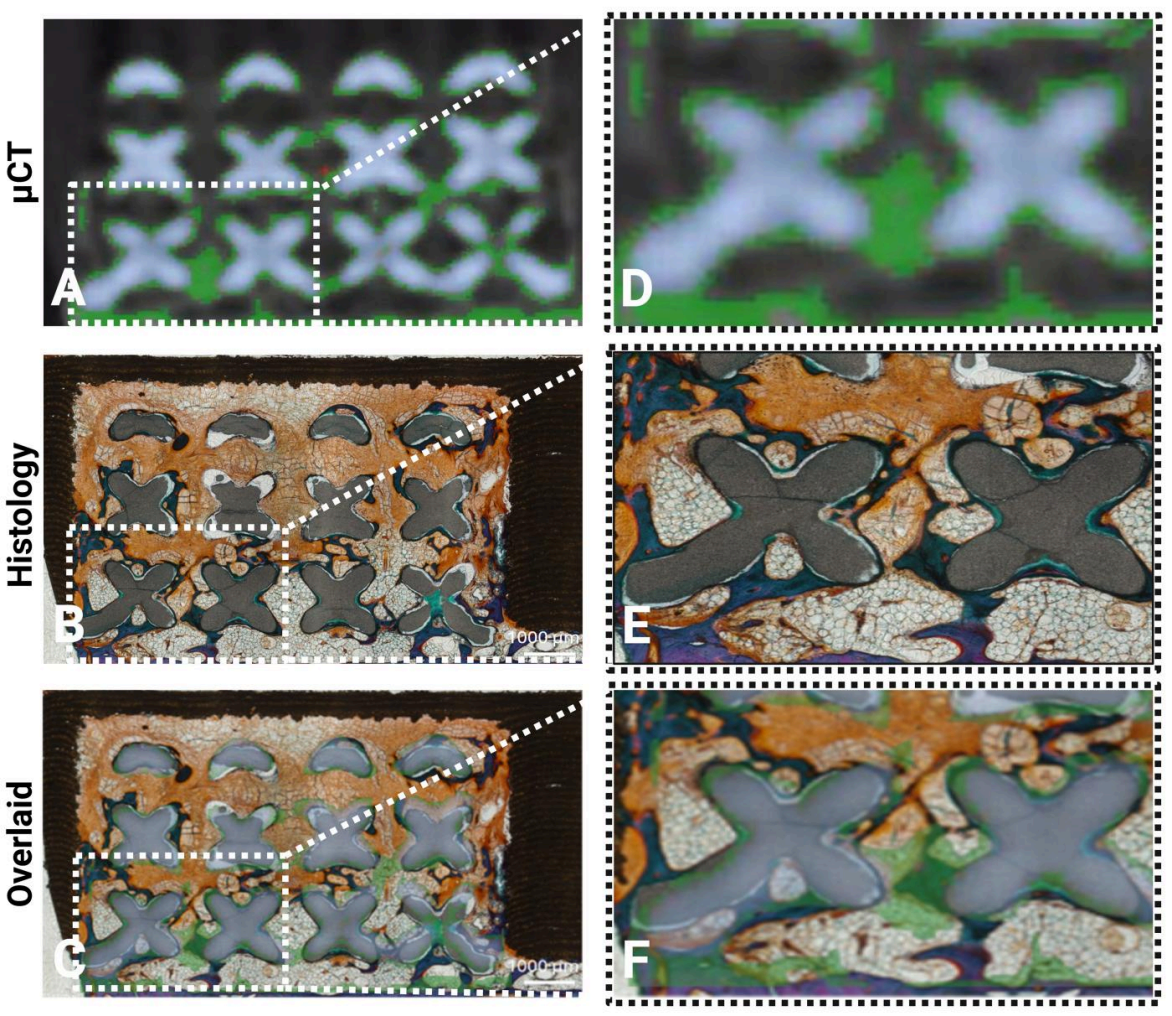
Large Pore bioreactor (lower chamber): μCT–histology comparison for mineralized tissue assessment. (**A**) μCT reconstruction of the Large Pore scaffold with segmented mineralized tissue (green) localized around scaffold struts. (**B**) Goldner’s trichrome-stained histological section of the same slice, showing mineralized bone (blue), osteoid (red–orange) and scaffold (grey). (**C**) Overlay of μCT and histology images for the same region. (**D–F**) Magnified views of the boxed region in **A–C**, enabling direct visual comparison of mineralized tissue distribution between modalities. Scale bars: 1000 μm.

Bioreactors representing high and low mineralized tissue content, as determined by end‑point 3D μCT analysis, were selected for histological staining to verify the μCT findings. Slice‑matched comparisons of μCT reconstructions and Goldner’s trichrome‑stained resin-embedded sections were performed, with μCT datasets re‑segmented to the exact histological plane for direct location‑matched assessment. Figures  5–7 provide overall representations of this verification process, illustrating typical μCT-histology for each group. Across all groups, overlays confirmed that μCT segmentation reliably identified high‑density mineralized tissue, but histology consistently detected additional low-density bone not captured at the applied threshold, illustrating both the strengths of μCT for volumetric quantification and its inherent limitations in detecting early or partially mineralized bone.

In the AUTG group, μCT (Figure  5A) revealed mineralization along the periphery of β‑TCP struts, with limited extension into central pore spaces. The corresponding Goldner’s trichrome‑stained section (Figure  5B) confirmed these regions as mature mineralized bone (shown in blue) integrated with the scaffold surface, interspersed with osteoid and fibrous tissue in non‑mineralized areas. The overlay image (Figure  5C) demonstrated precise alignment of scaffold geometry and mineralized regions between modalities. Magnified views of the highlighted region (Figure  5D–F) confirm spatial registration. Small foci of histologically apparent bone, visible in Figure  5E but absent in Figure  5D, likely represent lower‑density mineralized tissue with attenuation values below the applied μCT threshold (≥ 1400 HU).

In the Small Pore scaffolds, μCT reconstruction (Figure  6A) showed extensive mineralized tissue both along scaffold struts and bridging pore spaces. The corresponding histological section (Figure  6B) confirmed dense, well‑integrated bone (blue) within the scaffold and the overlay (Figure  6C) demonstrated strong spatial concordance between modalities. Magnified views (Figure  6D–F) revealed additional low‑density bone not captured by μCT due to thresholding constraints.

In the Large Pore scaffolds, μCT reconstruction (Figure  7A) showed patchy mineralization (green) mainly along scaffold struts. The corresponding histological section (Figure  7B) revealed additional regions of new bone (blue), including low-density tissue not detected by μCT. The overlay (Figure  7C) indicated that the two modalities mostly matched, with histology extending the distribution of mineralized tissue beyond what was segmented on μCT. Magnified views (Figure  7D–F) confirmed that μCT primarily captured high-density mineralized bone, while histology also identified immature or less mineralized matrix below the 1400 HU threshold. The greater discrepancy in this group likely reflects the larger pore size and more heterogeneous mineralization pattern.

## Discussion

This study investigated bone regeneration in a preclinical sheep model using µCT analysis, with additional histological verification to characterize and confirm new bone formation. We examined how scaffold structure, the presence and source of osteogenically differentiated ADSCs, and tissue contact within the implantation site affect outcomes. Bioreactor chambers incorporating SP-βTCP scaffolds, engineered with varying pore sizes and loaded with either autologous or allogeneic ADSCs, were surgically implanted onto the scapula. New bone formation was quantitatively assessed using µCT, while histological analysis not only verified the µCT findings but also provided complementary qualitative evidence of bone tissue organization. This enabled a detailed evaluation of how scaffold design and biological cues influence the spatial and qualitative characteristics of bone regeneration.

The μCT analysis demonstrated significantly greater bone formation in Small Pore scaffolds (0.66 mm) compared to Large Pore scaffolds (0.93 mm), highlighting the strong influence of pore size on bone regeneration. In bone tissue engineering, pore size represents the average diameter of scaffold voids and is critical for cell migration, vascularization, nutrient exchange, waste removal and overall tissue regeneration [27, 28]. At the microscale, pores <100 µm increase scaffold surface area, promote osteoconduction and enhance early matrix deposition [29, 30], while mesopores of 300–500 µm optimize cell infiltration, angiogenesis and nutrient diffusion [31, 32]. Although these findings relate to trabecular-scale or intra-strut porosity, the principles of surface area, interconnectivity and mass transport similarly apply to millimetre-scale architectures [33, 34]. Additionally, variations in scaffold geometry influence mechanical behaviour, fluid flow and cellular responses, leading to differences in bone formation even when nominal pore sizes are identical [28].

Fabricating scaffolds with sub-millimetre pores remains limited by current additive manufacturing capabilities. Printing such fine features in materials like βTCP often produces mechanically fragile structures prone to deformation, especially in load-bearing contexts [35]. Narrow struts and interconnections also complicate post-processing, as removing unfused material or supports can damage the scaffold [36]. For this reason, pore sizes in this study were selected to balance manufacturability, structural integrity and compatibility with secondary biomaterials such as hydrogels or cell-laden matrices. Because infilling reduces functional pore diameter, pores printed near the lower threshold for effective mass transport and cell migration may fall below critical limits, restricting permeability, nutrient diffusion and cellular infiltration [37, 38].

In our study, Small Pore scaffolds (0.66 mm) supported significantly greater bone formation than Large Pore scaffolds (0.93 mm), reinforcing how sensitive scaffold-guided regeneration is to relatively small changes in pore size. This aligns with reports that pores in the 400–800 µm range optimize osteogenesis and vascularization [38–40], whereas pores approaching or exceeding 1 mm reduce osteoconductive surface area and increase fibrous tissue infiltration [41]. These findings support a threshold effect in which modest increases beyond the optimal pore window shift the regenerative response away from mineralized tissue formation.

The µCT evaluation showed that autologous ADSCs (AUTG group) significantly increased bone volume in the lower bioreactors compared to GelMA alone (TG group; *P* < 0.01), supporting their osteogenic and pro-regenerative potential. While this is often attributed to ADSC differentiation and paracrine signalling [42], ADSCs may also indirectly enhance mineralization by promoting osteoclast-mediated βTCP resorption and calcium release [43, 44]. The synergistic interaction between ADSCs and βTCP may therefore contribute to increased bone formation through both cellular activity and scaffold-mediated mineral deposition [45, 46]. Close contact with native bone likely further supported vascularization and progenitor cell recruitment [47], as the cortical interface provides stable anchorage and communication with marrow-derived cells and vascular networks [48]. Additionally, the GelMA matrix may have modulated ADSC osteogenesis by providing arginine–glycine–aspartic acid (RGD)-mediated adhesion cues and stiffness-dependent mechanotransduction that regulate yes-associated protein/transcriptional co-activator with PDZ-binding motif (YAP/TAZ) and Runt-related transcription factor 2 (RUNX2) activity [49, 50].

The comparable BVs generated by autologous and allogeneic ADSCs are consistent with the recognized low immunogenicity of multipotent stromal cells [51]. Although not fully immune-privileged, their reduced MHC Class I expression enables partial immune evasion and supports tissue regeneration [52], while their secretion of anti-inflammatory cytokines helps modulate host immune responses and facilitate allograft acceptance without immunosuppression [53]. In this study, no clinical signs of local or systemic inflammation, such as swelling, fever, behavioural changes or graft rejection, were observed at any time, even after withdrawal of postoperative analgesics, supporting the *in vivo* compatibility of allogeneic ADSCs. Preclinical studies similarly report successful bone repair using allogeneic ADSCs across multiple models [54–56], reinforcing their potential as readily available, off-the-shelf cell therapies that overcome donor-related delays associated with autologous cell expansion. However, although no overt inflammatory response was observed clinically or histologically, further studies incorporating immune cell-specific markers and cytokine profiling are required to fully elucidate host–graft immune interactions.

In contrast to the lower bioreactors, no significant differences were observed in the upper bioreactors across any groups, suggesting inadequate or inconsistent periosteal contact. This likely stemmed from procedural challenges that compromised delivery of the periosteal cambium, a fragile layer whose osteogenic capacity depends heavily on vascular density and progenitor cell content, both of which vary with age, anatomical site and surgical technique [57–60]. Limited periosteal integrity would restrict angiogenic ingrowth and cellular recruitment, explaining the uniformly low regeneration in the upper chambers. By comparison, cortical bone provides a more stable and vascularized interface, with Haversian and Volkmann canal networks supporting reliable osteoconductive and osteoinductive signalling [61], consistent with the enhanced bone formation observed in the lower bioreactors. These findings align with reports emphasizing that periosteal-driven regeneration is highly dependent on robust angiogenesis and consistent progenitor cell recruitment [62, 63], reinforcing that the surrounding anatomical niche is an active determinant of scaffold-mediated regeneration rather than a passive boundary [64].

The comparative evaluation between µCT and histology assessed whether mineralized tissue identified by µCT corresponded to histologically confirmed bone. Overlaid images showed strong anatomical and morphological agreement, with µCT-classified mineralized regions matching bone-specific staining patterns in histological sections. This supports the validity of µCT for quantifying new bone formation, while recognizing that conservative segmentation thresholds likely underestimated early or low-density mineralization, an effect widely reported in the literature [65, 66]. Histology captured immature woven bone and partially mineralized matrix that fell below the 1400 HU threshold, revealing regenerative activity not detected by µCT. These observations indicate that scaffold orientation influenced not only total mineralized volume but also the establishment of early osteoid, and highlight the value of combining µCT with histology to fully characterize scaffold-mediated bone regeneration.

By capturing low-density or immature regions of mineralized matrix that µCT could not detect, histological analysis with Goldner’s trichrome provided important complementary insight into the regenerative process. Additionally, observations from the histological image analysis also support the overall outcomes demonstrated by the µCT, followed by the corresponding histological verification of new bone formation across the different groups in this study. This additional sensitivity showed that chamber orientation influenced not only the total volume of mineralized tissue but also the establishment of early osteoid. In doing so, histology extended the interpretation of the µCT data, demonstrating that periosteum-facing and native bone-facing configurations created distinct regenerative environments, with differences evident even below the µCT detection threshold. These findings highlight clear strategies for improving scaffold performance: tailoring pore size, architecture and chamber orientation to the biological interface can better support osteoconduction, vascularization and progenitor cell recruitment, which are critical for regenerating large bone defects.

### Limitations

The differences in access to osteogenic niches between the upper and lower bioreactor chambers likely contributed to the variation in bone formation observed. Furthermore, the study was also subject to individual biological variability among the sheep, which could have introduced heterogeneity in the extent and quality of bone regeneration observed. A further limitation of this study relates to the methodological differences between the two imaging approaches. Histology was performed to verify the μCT findings, using thin, stained tissue sections. For this verification, the μCT dataset was re-segmented in Imalytics to extract the single slice corresponding exactly to the histological section, enabling direct, location-matched overlay of the two modalities. By contrast, the primary μCT analysis was conducted on the entire 3D scaffold volume. This inherent difference between whole-volume μCT quantification and single-section histological assessment may account for the minor variability observed between the modalities.

Another limitation of this study is that the histological image analysis was performed only on a subset of Goldner’s trichrome-stained sections selected for µCT verification, rather than across all harvested samples. Because the analysis was restricted to spatially matched representative tissue sections rather than the full scaffold volume, the dataset was not sufficiently powered for formal statistical comparison and should therefore be interpreted as descriptive, supportive evidence only.

## Conclusion

This study highlights that successful bone regeneration results from the combined influence of scaffold design, cell source and anatomical context rather than any single factor. By demonstrating that scaffold pore architecture and chamber orientation shape the regenerative microenvironment, and that both autologous and allogeneic ADSCs support new bone formation, our findings emphasize the importance of integrating structural optimization with biologically relevant cell strategies. While autologous ADSCs generally exhibited superior osteogenic outcomes, the comparable regenerative performance observed with allogeneic ADSCs indicates that both cell sources can be efficacious. This suggests that allogeneic ADSCs hold strong translational promise as a practical, off-the-shelf alternative to autologous cells, offering scalability and broader clinical applicability in bone tissue engineering.

## Supplementary Material

rbag097_Supplementary_Data
