# Supplementary material for: Evaluation of bone formation within β-tricalcium phosphate scaffolds in a sheep scapular bioreactor model using micro-computed tomography analysis
Source: Regen Biomater. 2026 May 21;13:rbag097. doi: 10.1093/rb/rbag097 (PMC13275134; doi:10.1093/rb/rbag097)
Supplement: rbag097_Supplementary_Data [file rbag097_supplementary_data.docx]

**Supplementary Data**

**Histological Image Analysis**

Semi-quantitative histological image analysis of the matched verification sections generally supported the trends observed in the µCT-based assessment of new bone formation. Across all comparisons, greater bone area (BA) and BA/TA values were predominantly observed in the lower chambers relative to the upper chambers, indicating more extensive bone formation in constructs positioned closer to the native scapular surface.

In the pore architecture comparison **(Supplementary Figure 2A and 2D)**, the small pore SP-βTCP scaffolds exhibited the highest histologically measured bone area in the lower chamber, exceeding that of the large pore scaffolds. This trend was similarly reflected in the BA/TA ratio, where the small pore group demonstrated a greater proportion of bone occupancy within the analysed tissue area. In the upper chamber, however, the opposite trend was observed, with the large pore scaffolds showing greater bone area and BA/TA values than the small pore group, although bone formation in the upper chamber remained comparatively limited overall.

In the TG versus AUTG comparison **(Supplementary Figure 2B and 2E)**, the AUTG lower chamber showed a greater bone area and BA/TA ratio than the TG group, consistent with more extensive histologically evident bone formation in the autologous ADSC-loaded constructs. In contrast, within the upper chamber, the TG group exhibited greater bone area and BA/TA values than AUTG, while the AUTG upper chamber demonstrated minimal bone presence in the matched section analysed.

Similarly, in the AUTG versus ALTG comparison **(Supplementary Figure 2C and 2F)**, the AUTG lower chamber showed substantially greater bone area and BA/TA values than the ALTG group. In the upper chamber, both groups exhibited very limited bone formation, with only negligible bone area detected histologically.
